# Case Report: Gollop-Wolfgang Complex in a 5 month old baby

**DOI:** 10.12688/f1000research.5889.3

**Published:** 2015-02-24

**Authors:** Ihtesham A. Qureshi, Rohit Kumar Gudepu, Ravikanth Chava, Sravya Emmani, Syed Husain Asghar, Mohtashim A. Qureshi, Nimmathota Arlappa

**Affiliations:** 1 Division of Community Studies, National Institute of Nutrition, Indian Council of Medical Research, Hyderabad, 500 007, India

**Keywords:** Skeletal dysplasias, Gollop-Wolfgang complex, limb deficiency

## Abstract

Skeletal dysplasias are disorders associated with a generalized abnormality in the skeleton. The Gollop-Wolfgang complex (GWC) is a limb deficiency disorder and an unusual limb malformation with highly variable manifestations. Here we report an interesting case of a 5-month old male baby from India with Gollop-Wolfgang Complex showing bifurcation of the right femur, ectrodactyly of both feet, ectrodactyly of left hand, syndactyly of right hand and unusual presentation of bilateral fibular agenesis and caudal (Sacrococcygeal) agenesis. The etiology of GWC in this 5 month old male baby could possibly be attributed to spontaneous gene mutation. The clinical, radiographic findings and the unusual presentation are presented in detail.

## Introduction

Generalized disorders of cartilage and bone have been referred to as skeletal dysplasias and are associated with a generalized abnormality in the skeleton ^1^. Gollop-Wolfgang Complex (GWC) is a rare congenital limb anomaly characterized by tibial aplasia, ipsilateral bifurcation of the thighbone and ectrodactyly ^2^. Ectrodactyly involves the deficiency or absence of one or more central digits of the hand or foot and is also known as split hand/split foot malformation (SHFM) ^3^. Very often, the anomalies of limbs, heart, digestive and urinary tracts and the lumbosacral vertebrae are also affected ^4^.

In 1980, Gollop *et al.* described the case two brothers with ectrodactyly and unilateral bifurcation of the femur, absence of both tibiae and monodactyly of the feet ^5^. In 1984, Wolfgang reported a case of right femoral bifurcation and absence of tibia and bilateral central defects of the hand ^5^. Lurie and Ilyina (1986) proposed the eponym GWC for the combination of femoral bifurcation with hand ectrodactyly ^6^. Endo *et al.* found a total of 12 reported cases and added the case of a Japanese girl with a unique form of this malformation complex. Both hands and feet were involved and the involvement was bilateral ^2^. The etiology of GWC is most likely an error in the complex genetic control of limb development but the exact cause is still unclear ^7^. GWC is listed as a “rare disease” by the United States Office of Rare Diseases [ORD] of the National Institute of Health [NIH] and the approximate incidence is 1 in 1000,000 ^8^.

## Case presentation

**Figure 1.**
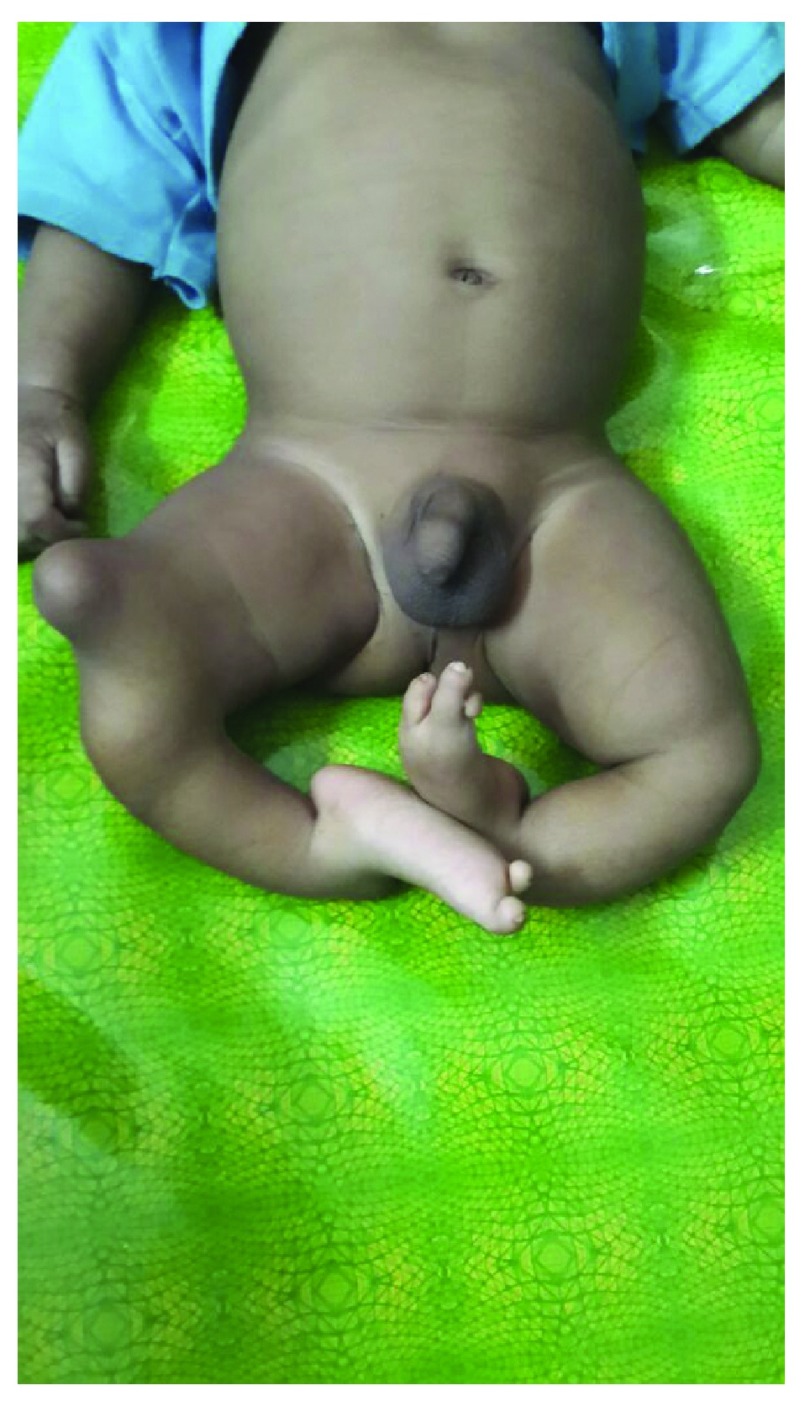
Limb deformities.

**Figure 2.**
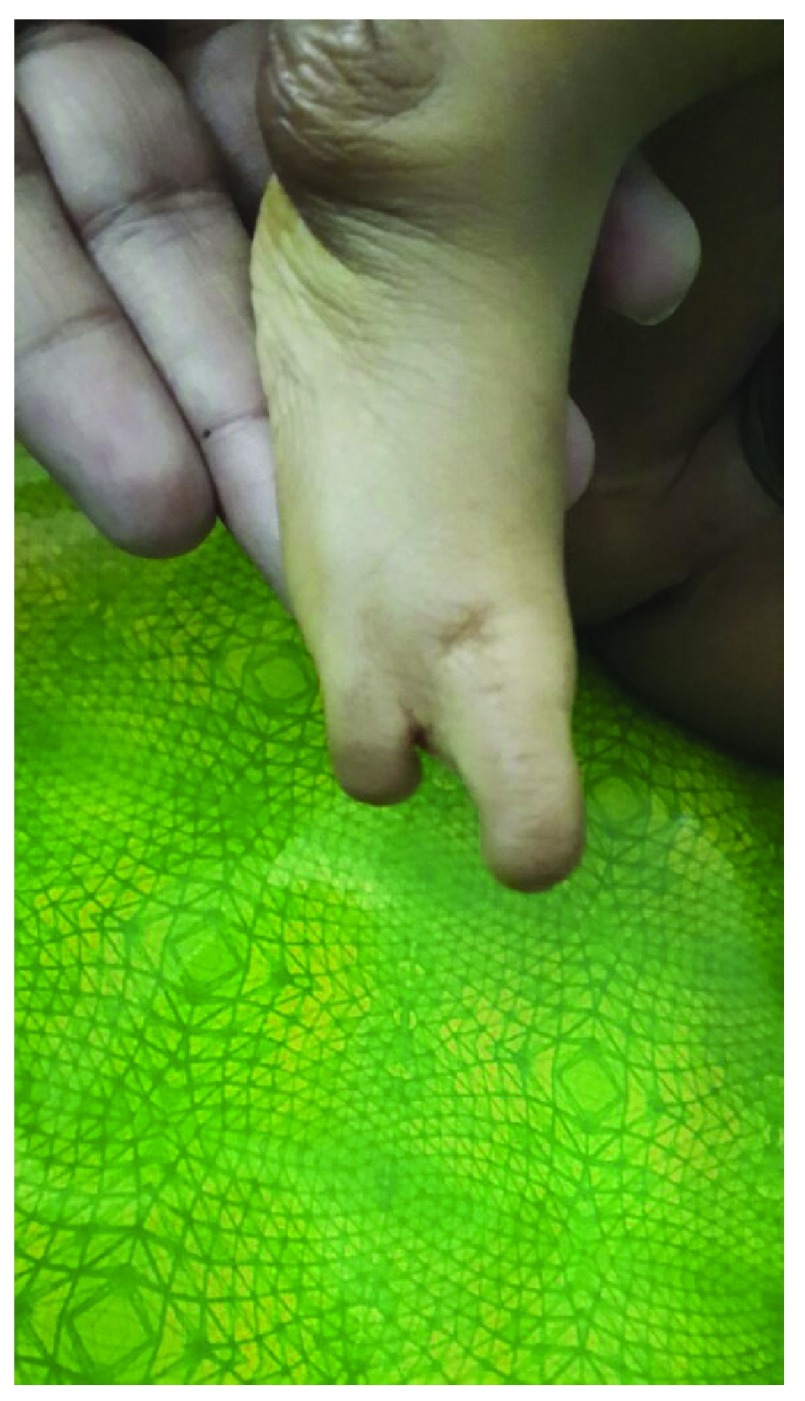
Ectrodactyly of toes.

**Figure 3.**
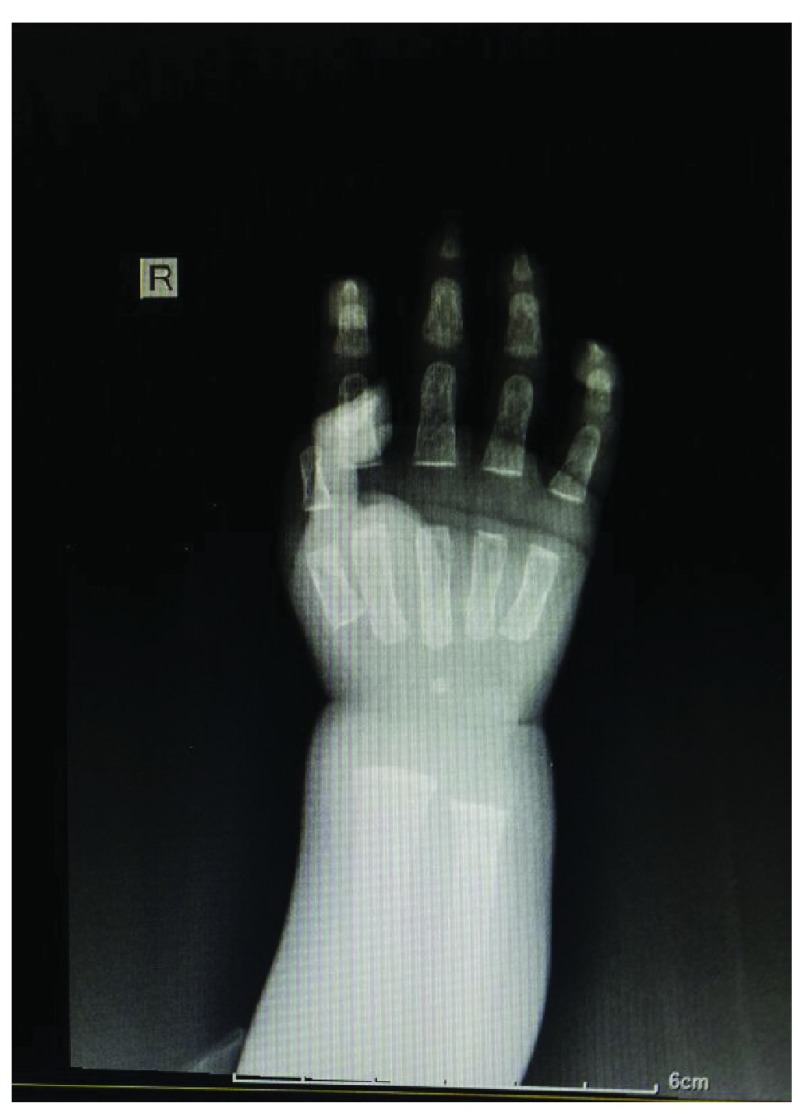
X-ray right hand showing syndactyly.

**Figure 4.**
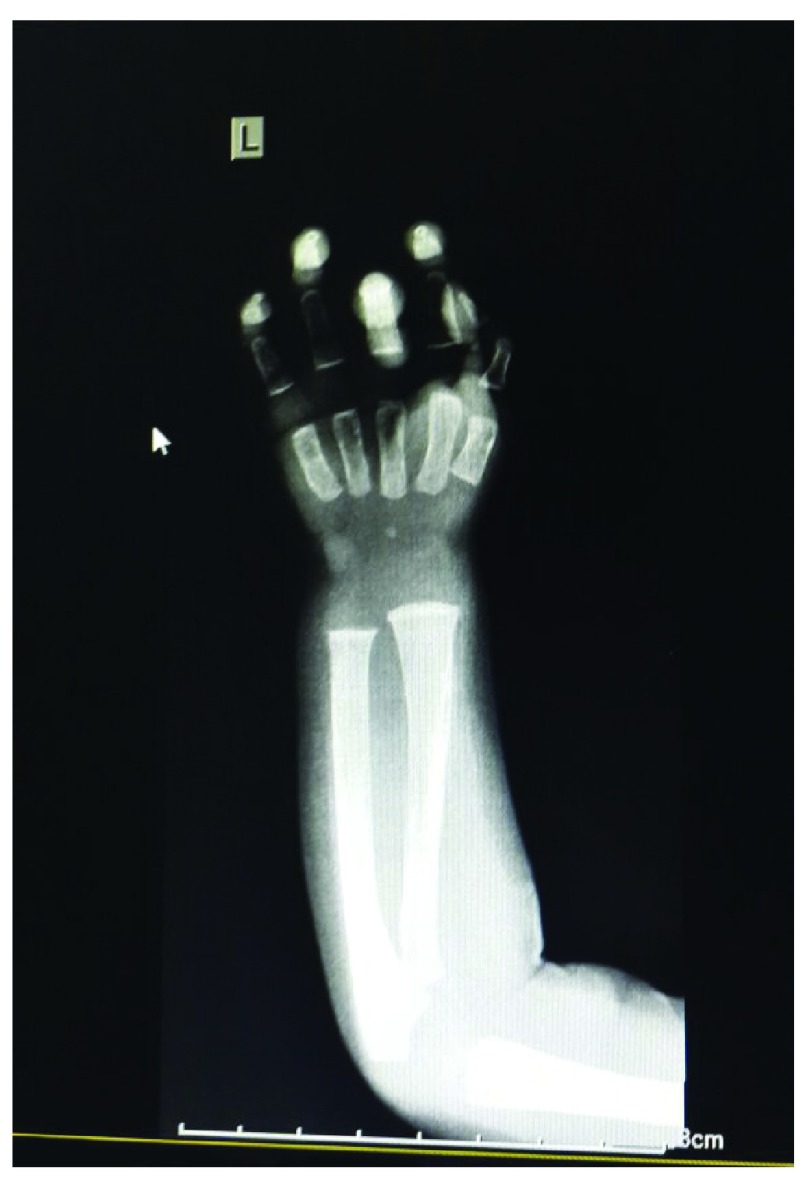
X-ray of left hand showing ectrodactyly.

**Figure 5.**
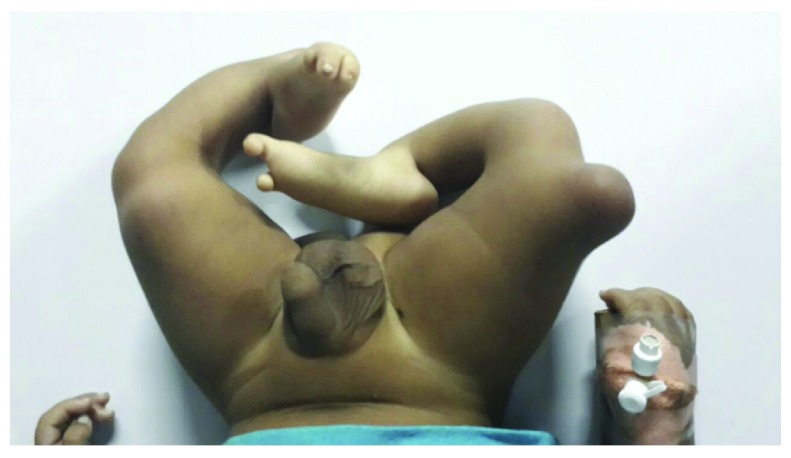
Protrusion over right thigh.

**Figure 6.**
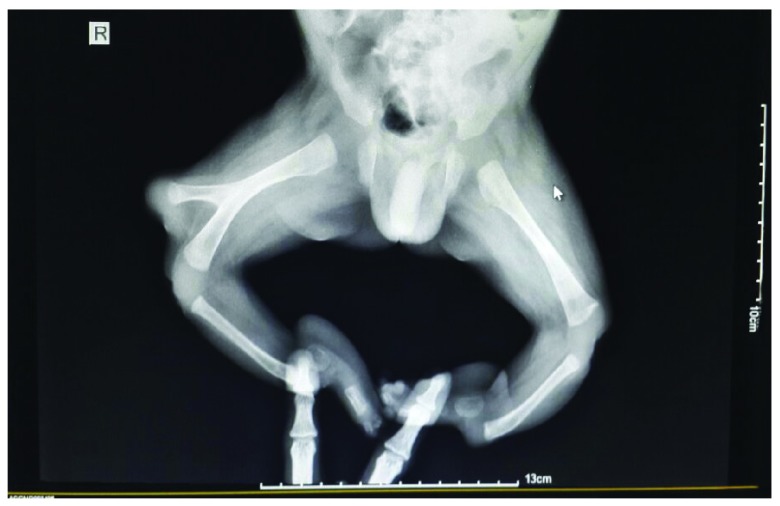
X-ray showing bifid femur with fibular agenesis.

**Figure 7.**
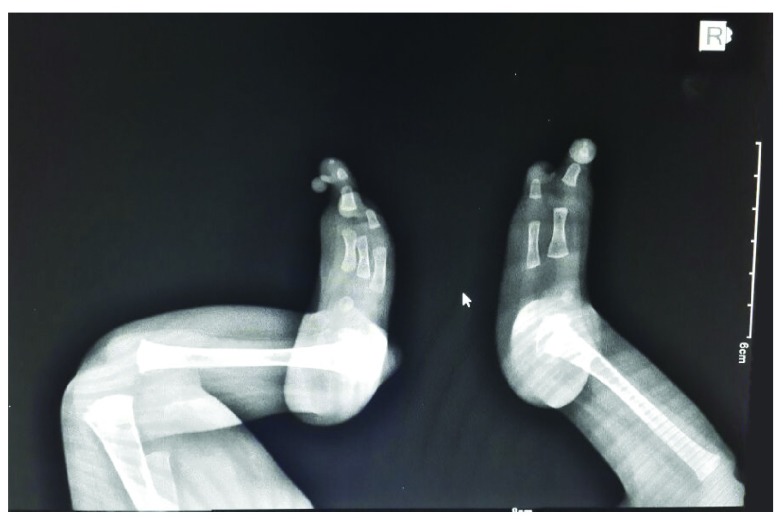
X-ray showing absence of right 3,4,5 metatarsals and phalanges, absence of left 4,5 metatarsals and phalanges.

**Figure 8.**
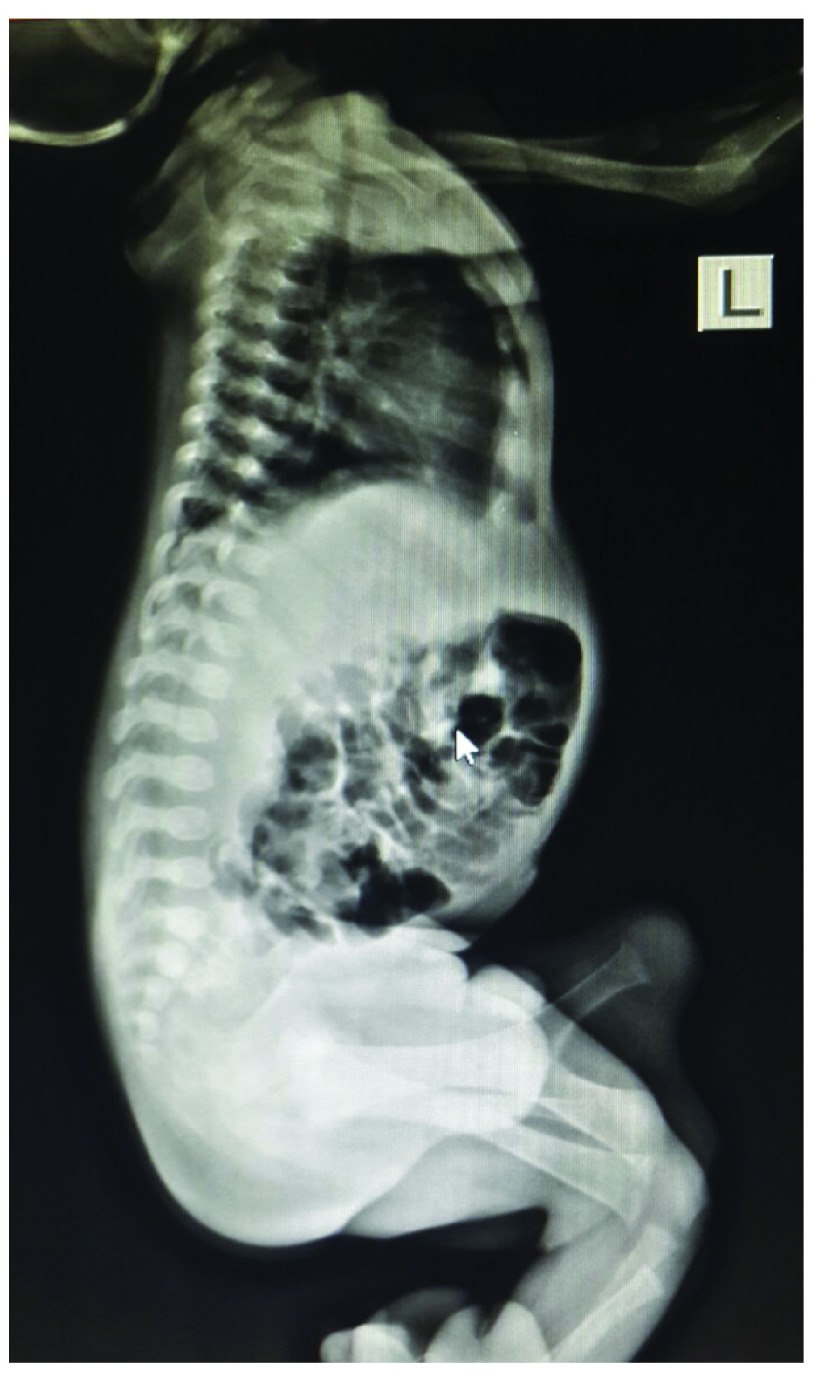
X-ray showing caudal (sacrococcygeal) agenesis.

A 5-month old male Indian child with normal karyotype (46 XY) born to a 26-year-old primigravida, full term by C-section, presented with limb deformities associated with bilateral ectrodactyly of feet ( Figure 1 and Figure 2), syndactyly of right hand ( Figure 3) and ectrodactyly of left hand ( Figure 4). At the medial distal third of the right femur, a large protrusion was present ( Figure 1 and Figure 5). Radiographic images showed bifid femur with fibular agenesis ( Figure 6), absence of right 3, 4, 5 metatarsals and phalanges, absence of left 4, 5 metatarsals and phalanges of foot ( Figure 7), left lateral X-ray showing caudal (sacrococcygeal) agenesis ( Figure 8). Initial diagnosis was made when the parents brought the child to the out-patient department concerned about limb abnormality at the age of 3 months and the final diagnosis was made following admission to the in-patient unit at 5 months, based on both clinical presentation and radiological images. There was no detailed prenatal history available.

The parents had documented second degree consanguinity but both did not have any significant family history. Similarly, there was no history of exposure to radiation, prenatal teratogenic medications and infections during pregnancy. The mother did not smoke or drink during pregnancy. The child was breast-fed with good appetite and cry, without any bowel bladder problems, change in skin color or any cleft lip/palate. Echocardiography at the time of admittance revealed no congenital heart defects. The ultrasonography of abdomen and pelvis revealed no visceral or renal abnormalities. Surgical reconstruction treatment was advised but the parents did not give consent for treatment.

## Discussion

One case was reported of an Arab Muslim couple who came from a region where other consanguineous families with similarly affected individuals had been reported Kohn *et al.* in 1989 ^9^, and the autosomal recessive inheritance seemed evident in the case of a child described by Raas-Rothschild *et al.* in 1999 ^10^. In this case, we report a typical presentation of GWC with bilateral fibular agenesis and sacrococcygeal agenesis along with pathognomonic features of GWC (bifurcation of femur, syndactyly and ectrodactyly). There were no associated abnormalities like cleft lip/palate, tibial agenesis, visceral or cardiac anomalies seen in this patient. There is one documented case reported with distal femoral duplication with fibular agenesis ^11^. The best treatment option for this patient with GWC is early knee disarticulation and resection of the protruded bifurcated femur, followed by fitting of a modern prosthesis ^12^. This treatment was discussed with the parents of the patient at three months of age and a follow-up visit was scheduled after two months.

History of consanguinity is strongly associated with the developments of congenital anomalies among the newborn babies; there should be pre-marital genetic counselling to evaluate any impending congenital abnormalities. Similarly, antenatal check-ups are appropriate for early detection of congenital anomalies through proper screening.

## Consent

Informed written consent for publication of images and clinical details was obtained from the patient’s parents.
