# Supplementary material for: Case Report: Gollop-Wolfgang Complex in a 5 month old baby
Source: F1000Res. 2015 Feb 24;3:315. Originally published 2014 Dec 23. [Version 3] doi: 10.12688/f1000research.5889.3 (PMC4448748; doi:10.12688/f1000research.5889.3)
Supplement: Peer review history

# Peer review: Referee response for version 3

**DOI:** 10.5256/f1000research.6622.r8336

**Published:** 2015-05-19

**Authors:** Murat Bastepe, Qing He

**Affiliations:** 1 Endocrine Unit, Massachusetts General Hospital, Boston, MA, 02114, USA; 2 Endocrine Unit, Massachusetts General Hospital, Boston, MA, USA

This report describes a 5-month-old patient with rare Gollop-Wolfgang Complex (GWC) disorder.  The patient exhibits typical phenotypes of GWC disease, such as ectrodactylyl of the hand and bifurcation of the femur.

The etiology of GWC remains unknown. However, the patient’s parents are second degree relatives, and other reports have described GWC patients in consanguineous families, suggesting that this disease is inherited in an autosomal recessive manner.

The manuscript is well-written, and I suggest it is indexed in current form.

We have read this submission. We believe that we have an appropriate level of expertise to confirm that it is of an acceptable scientific standard.

# Peer review: Referee response for version 3

**DOI:** 10.5256/f1000research.6622.r8149

**Published:** 2015-03-30

**Authors:** Nicole Philip

**Affiliations:** 1 Département de Génétique Médicale et de Biologie Cellulaire, Assistance Publique - Hopitaux de Marseille, Marseille, France

I have read this submission. I believe that I have an appropriate level of expertise to confirm that it is of an acceptable scientific standard.

# Peer review: Referee response for version 2

**DOI:** 10.5256/f1000research.6552.r7661

**Published:** 2015-02-13

**Authors:** Nicole Philip

**Affiliations:** 1 Département de Génétique Médicale et de Biologie Cellulaire, Assistance Publique - Hopitaux de Marseille, Marseille, France

I fully agree with the first reviewers's comments.

This paper deserves indexing as it is a very rare disorder. However, the following sentence (in the discussion) should be deleted:

"This type of skeletal dysplasia with limb deficiencies could be the result of spontaneous gene mutations and chronic exposure to a toxic substance or infectious agents that results in the disruption of normal skeletal development."

The main conclusion of this report should be that the occurrence of Gollop-Wolfgang in a consanguineous family brings further arguments to support autosomal recessive inheritance.

I have read this submission. I believe that I have an appropriate level of expertise to confirm that it is of an acceptable scientific standard.

# Peer review: Referee response for version 1

**DOI:** 10.5256/f1000research.6291.r7449

**Published:** 2015-01-27

**Authors:** Stephen Robertson

**Affiliations:** 1 Department of Genetics, Dunedin School of Medicine, University of Otago, Dunedin, New Zealand

This report describes a child with a typical presentation of Wolfgang-Gollop complex. The eitiology of this condition is still in doubt although some familial recurrences suggest a genetic cause. The statement in the abstract that a spontaneous mutation is possible owing to parental consanguinity does not make sense and should be deleted. In this discussion there is also a suggestion that consanguineous unions should somehow be discouraged. This is inappropriate and a breach of reproductive autonomy. Such a statement should be removed. Consanguinity is not the strong risk factor for congenital anomalies as the authors imply, conferring approximately a 2 fold enhanced risk of such problems.  This assertion needs to be rebalanced.

I have read this submission. I believe that I have an appropriate level of expertise to confirm that it is of an acceptable scientific standard, however I have significant reservations, as outlined above.

# Response

**Published:** 2015-02-02

**Authors:** aatif qureshi

Really appreciate your comments Dr. Stephen Robertson. I would like to hear back about my newer version of the paper.
